# Transcriptomic Analysis of *MSTN* Knockout in the Early Differentiation of Chicken Fetal Myoblasts

**DOI:** 10.3390/genes13010058

**Published:** 2021-12-26

**Authors:** Ke Xu, Hao Zhou, Chengxiao Han, Zhong Xu, Jinmei Ding, Jianshen Zhu, Chao Qin, Huaixi Luo, Kangchun Chen, Shengyao Jiang, Jiajia Liu, Wenqi Zhu, He Meng

**Affiliations:** 1 Shanghai Collaborative Innovation Center of Agri-Seeds, School of Agriculture and Biology, Shanghai Jiao Tong University, Shanghai 200240, China; keristina@sjtu.edu.cn (K.X.); zhouhao1992@sjtu.edu.cn (H.Z.); rex_han@sjtu.edu.cn (C.H.); dingjinmei@sjtu.edu.cn (J.D.); zhujianshen@sjtu.edu.cn (J.Z.); qc515150910075wymq@sjtu.edu.cn (C.Q.); huangjingxiang@sjtu.edu.cn (H.L.); chenkangchun@sjtu.edu.cn (K.C.); aimeegirl@sjtu.edu.cn (S.J.); jiajialiu@sjtu.edu.cn (J.L.); zwqjssz@sjtu.edu.cn (W.Z.); 2 Hubei Key Laboratory of Animal Embryo and Molecular Breeding, Institute of Animal Husbandry and Veterinary, Hubei Provincial Academy of Agricultural Sciences, Wuhan 430072, China; xz8907@163.com

**Keywords:** *Myostatin*, CRISPR/Cas9, transcriptome, skeletal muscle, growth and development

## Abstract

In mammals, *Myostatin (MSTN)* is a known negative regulator of muscle growth and development, but its role in birds is poorly understood. To investigate the molecular mechanism of *MSTN* on muscle growth and development in chickens, we knocked out *MSTN* in chicken fetal myoblasts (CFMs) and sequenced the mRNA transcriptomes. The amplicon sequencing results show that the editing efficiency of the cells was 76%. The transcriptomic results showed that 296 differentially expressed genes were generated after down-regulation of *MSTN*, including *angiotensin I converting enzyme* (*ACE)*, *extracellular fatty acid-binding protein* (*EXFABP)* and *troponin T1, slow skeletal type (TNNT1).* These genes are closely associated with myoblast differentiation, muscle growth and energy metabolism. Subsequent enrichment analysis showed that DEGs of CFMs were related to MAPK, Pl3K/Akt, and STAT3 signaling pathways. The MAPK and Pl3K/Akt signaling pathways are two of the three known signaling pathways involved in the biological effects of *MSTN* in mammals, and the STAT3 pathway is also significantly enriched in *MSTN* knock out chicken leg muscles. The results of this study will help to understand the possible molecular mechanism of *MSTN* regulating the early differentiation of CFMs and lay a foundation for further research on the molecular mechanism of *MSTN* involvement in muscle growth and development.

## 1. Introduction

The chicken has been used as a model research organism for many years, and it also provides large amounts of protein for the human diet [1]. In the domestic poultry industry, increasing meat production is an important goal; therefore, a full understanding of the regulatory mechanisms of muscle proliferation and differentiation is needed for improvement of growth and meat production traits [2]. However, knowledge is limited regarding the molecular mechanisms underlying muscle growth and development in birds in general, and in broiler chickens in particular.

*Myostatin* (*MSTN*), also known as a growth factor and differentiation factor-8 (*GDF-8*), is mainly expressed in skeletal muscle cells [3], and it acts as an essential negative regulator of skeletal muscle growth in livestock [4]. *MSTN* was first discovered and confirmed as a negative regulator of muscle growth in mice [3], and its inhibitory effect was subsequently further confirmed in cattle, pigs, chickens, goats, and humans [5,6,7,8,9,10]. Previous studies in mice and other mammals have shown that *MSTN* exerts its biological effects through multiple signaling pathways, including the mitogen-activated protein kinase (MAPK) signaling pathway [11], TGF-β [12], and the Pl3K/Akt [13] signaling pathway. Unlike the case for mammals, however, the functional and molecular mechanisms of *MSTN* in chicken muscle have not been well studied. 

The limited research on poultry has mainly focused on statistical genomic methods that have examined correlations between genomic SNPs or mutations in the *MSTN* gene and growth traits [14,15]. Extending these types of studies to include functional genomics has been difficult because gene knockout in chickens or other birds is difficult to perform due to differences in the avian reproductive system and embryonic development [16]. Therefore, few experimental results related to the developmental effects of *MSTN* are available for chickens. 

The development of skeletal muscle is determined by myogenesis, and the number of muscle fibers formed mainly depends on the degree of myoblast proliferation and differentiation [17]. Researchers have used CRISPR/Cas9 technology to knock out the *MSTN* gene in mouse myoblasts (C2C12 cells) and performed RNA-Seq and miRNA-Seq to explore the molecular mechanism of *MSTN* regulation of muscle cell proliferation [18]. Other studies have used CRISPR/Cas9 technology to generate *MSTN* knockout (KO) avian (quail) myoblasts (QM7) to examine the *MSTN* regulatory networks through RNA-Seq [19]. 

In the current study, we used the CRISPR/Cas9 system to knock out the *MSTN* gene in chicken fetal myoblasts (CFMs), which—like C2C12 and QM7 cells—are an important model for studying the molecular mechanisms of muscle growth and development [20,21]. Then we performed RNA-Seq technology on knockout (KO) and wild-type (WT) CFMs and used PCA analysis (a mathematical algorithm to reduce data complexity) to evaluate the similarities and differences between samples and determine that the samples can be grouped [22]. Finally, we screened *MSTN*-related genes, pathways, and biological processes in knockout (KO) vs. wild-type (WT) CFMs. Our research provides valuable evidence for the possible molecular mechanism by which *MSTN* regulates the early differentiation of CFMs and lays a foundation for further research on the molecular mechanism of *MSTN* involvement in muscle growth and development.

## 2. Materials and Methods

### 2.1. Plasmid Construction

*MSTN* exon 1 single guide RNAs (sgRNAs) were designed using http://chopchop.cbu.uib.no accessed on 11 May 2020. An adenoviral (AdV) vector co-expressing EGFP, SpCas9, and a pair of sgRNA was named AdV-EGFP-CRISPR system (Figure S1A). An AdV vector co-expressing EGFP was named AdV-EGFP system (Figure S1B). The two systems were custom designed by VectorBuilder.

### 2.2. Cell Culture

Specific pathogen-free (SPF) chicken eggs were obtained from Sais Poultry Co. Ltd., Jinan, China, and incubated at 37.8 °C under a relative humidity of 60%. 

Chicken fetal myoblasts (CFMs) were isolated from chicken embryos (*n* = 20) at 13 days of age and minced in 0.2% collagenase type I (Solarbio, Beijing, China) for digestion. The resulting suspension was dispersed, filtered, centrifuged, and plated in cell culture plates. The cultures were enriched in myoblasts by placing the cells in an incubator twice for 40 minutes to remove fibroblasts. The enriched culture was grown in DMEM/F12 medium (Gibco, Shanghai, China) supplemented with 20% FBS (Gibco, Shanghai, China) at 37 °C 5% CO_2_. The CFMs were induced to differentiate by replacing the growth medium containing 20% fetal bovine serum with differentiation medium containing 2% horse serum.

As non-muscle control cells, primary chicken embryonic fibroblasts (CEFs) were isolated from chicken embryos (*n* = 8) at 9 days of age. The heads, wings, legs, and abdominal organs were removed from the chicken embryos, and the remaining tissues were minced and digested with 0.25% trypsin (Gibco, Shanghai, China). The resulting suspension was filtered, centrifuged, and plated in cell culture plates. CEFs were grown in DMEM/F12 medium (Gibco, Shanghai, China) supplemented with 5% FBS (Gibco, Shanghai, China) at 37 °C 5% CO_2_.

### 2.3. Immunofluorescence

Only primary myoblasts were used for immunofluorescence identification. Primary myoblasts were fixed with 4% formaldehyde (Solarbio, Beijing, China) for 30 min and then washed three times in phosphate-buffered saline (PBS) (Gibco, Shanghai, China). The fixed cells were then permeabilized with 0.2% Triton X-100 (Solarbio, Beijing, China) for 15 min and then washed three times in PBS. The cells were blocked with goat serum (Solarbio, Beijing, China) for 30 min and incubated with anti-desmin (Bioss, Beijing, China) overnight at 4 °C. The cells were then incubated with goat anti-rabbit IgG/FITC (Bioss, Beijing, China) for 1 h at 37 °C. The cell nuclei were visualized using DAPI staining solution.

### 2.4. Cell Transduction

Gently drop the AdV-EGFP system and the AdV-EGFP-CRISPR system into a six-well plate containing chicken CFMs, with a confluence of 50–70%. The cells were transduced at a multiplicity of infection (MOI) of 1000, with the addition of 5 μg/mL polybrene. After incubation for 12h, the cells were gently washed twice with PBS, and fresh growth medium was added. The same experiment was also performed on CEFs. One day after transduction, the growth medium of CFMs was replaced with differentiation medium so that the primary myoblasts began to differentiate. The CEFs were again cultured in growth medium. Two days after transduction, the cells were evaluated by fluorescence microscopy under appropriate excitation filters and cell samples were collected for subsequent analysis.

### 2.5. Detection of Editing Effect and Efficiency by Amplicon Sequencing

Two days after transduction, genomic DNA was isolated for PCR using the following primers: Primer F: CTGGATGGCAGTAGTCAGCC; and Primer R: CTGTTGGGAGAGCCTGAGAA. PCR conditions were: 95 °C for 3 min; 35× (95 °C for 15 s, 56 °C for 15 s, 72 °C for 36 s); 72 °C for 5 min; followed by 4 °C. PCR products were shown on a 1% agarose gel. Experiment-specific barcodes were added to the 5’ end of the primer sequence (Table S1) and performed PCR amplification. The resulting PCR products were pooled and sequenced with 250 bp paired end reads on a NovaSeq instrument. The samples were demultiplexed according to the assigned barcode sequences. Data were analyzed using the CRISPResso2 software. The alignment of reads at the cleavage site was further analyzed.

### 2.6. Detection of Gene Expression by Transcriptome Sequencing

Two days after transduction of the AdV-EGFP system and the AdV-EGFP-CRISPR system, the total RNA of CFMs and CEFs were extracted by using TRIzol reagent (Invitrogen, Carlsbad, CA, USA), and transcriptome sequencing was performed. The concentration and quality of the extracted RNA were measured using a NanoDrop 2000 instrument (Thermo Scientific, Waltham, MA, USA) and an Agilent 2100 (Agilent, Santa Clara, CA, USA) instrument. The mRNA purification, library preparation, and sequencing steps were all performed at Shanghai Personal Biotechnology Company (Shanghai, China). A total of 12 samples (three replicates for each group) were sequenced on the Illumina Novaseq 6000 platform. 

We also performed real-time PCR and chose the β-actin gene as reference house-keeping gene. ChamQ Universal SYBR qPCR Master Mix (Vazyme, Nanjing, China) was used to perform the qPCR reactions in a Bio-rad CFX Connect Real-Time PCR System, with a 20 μL reaction system comprising 10 μL of ChamQ Universal SYBR qPCR Master Mix (2×), 0.4 μL of each of the forward and reverse primers (10 μM), 2 μL of cDNA and 7.2 μL of distilled water. The primers for *MSTN* were 1F: GCTTTTGGATGGGACTGGATT, and 1R: CAGGTGAGTGTGCGGGTATTT. The primers for β-actin were 2F: GAGAAATTGTGCGTGACATCA, and 2R: CCTGAACCTCTCATTGCCA. The program was 95 °C for 30 s; followed by 40 cycles of 95 °C for 10 s, 60 °C for 30 s; and ended with a melting curve analysis. The 2^−ΔΔCt^ method was used to calculate the relative expression levels between the KO and WT groups. Statistically significant differences between groups were calculated by Student’s t-test, and *p* < 0.05 was considered statistically significant.

### 2.7. Principal Component Analysis (PCA) and Differentially Expressed Gene (DEG) Analysis

The raw data were filtered using a quality control analysis, and clean reads were obtained by removing low quality reads. The clean reads were mapped to the Gallus gallus genome (GRCg6a) using HISAT2 (http://ccb.jhu.edu/software/hisat2/index.shtml accessed on 21 May 2021). We used DEGseq2 R package to perform PCA on each sample based on the expression level to identify underlying stratification across samples. Differentially expressed genes (DEGs) were analyzed using the same R package. Genes with a *p*-value < 0.05 and fold-change ≥2 or ≤0.5 were considered differentially expressed.

### 2.8. Enrichment Analysis of Differentially Expressed Genes (DEGs)

Gene Ontology (GO) enrichment analysis and Kyoto Encyclopedia of Genes and Genomes (KEGG) enrichment analysis of the DEGs were performed using the DAVID 6.8 Functional Annotation Tool (https://david.ncifcrf.gov/ accessed on 27 June 2021). Ingenuity Pathway Analysis (IPA) (Ingenuity Systems, Qiagen, California) software was employed to identify canonical pathways affected by DEGs and to determine the molecules and network interactions with *MSTN* by uploading DEGs and *MSTN*. In all analyses, the *p*-value < 0.05 was considered significantly different.

## 3. Results

### 3.1. Generation of CRISPR/Cas9-Mediated MSTN Knockout in Chicken Cells

**Figure 1:**
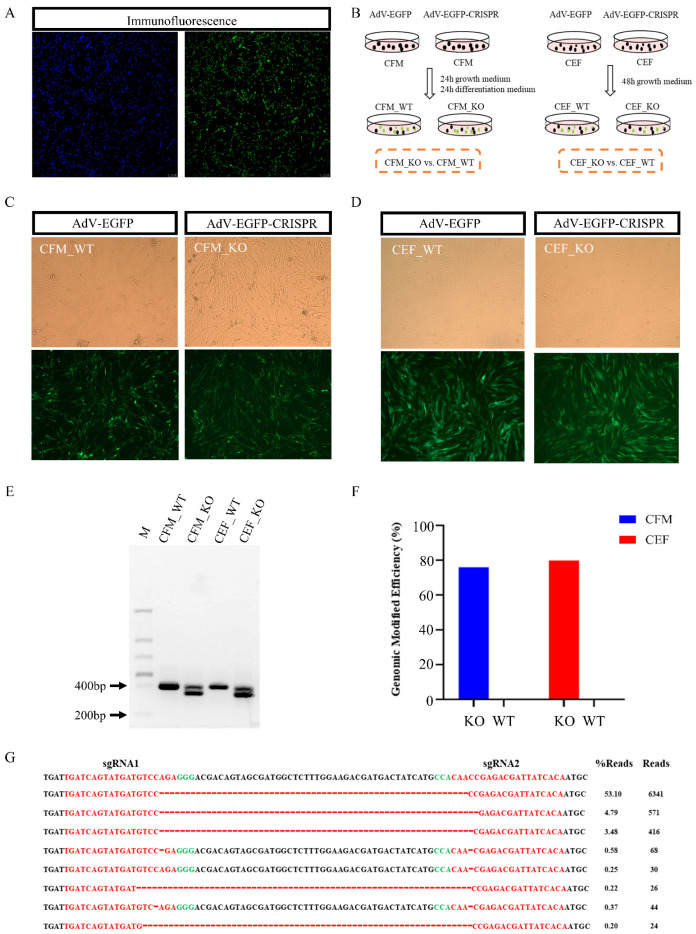
Preparation and validation of *MSTN* knockout (KO) cells. (**A**) Chicken fetal myoblasts (CFMs) were fixed, nuclei were stained with DAPI, and the cells were immunostained for desmin. (**B**) The experimental design was employed to knockout (KO) *MSTN* in CFMs and chicken embryonic fibroblasts (CEFs) and to produce the wild-type (WT) cells. Cells were transfected with the two viruses, and the cells were collected for transcriptome analysis after 48 hours. (**C**) Fluorescence (lower) and phase contrast (upper) microscopy images of CFMs transfected with AdV-EGFP and AdV-EGFP-CRISPR virus 48 hours after transfection. (**D**) Fluorescence (lower) and phase contrast (upper) microscopy images of CEFs transfected with AdV-EGFP and AdV-EGFP-CRISPR virus 48 hours after transfection. (**E**) Electrophoretogram of the results of all transfected cells validation by PCR. (**F**) The overall proportion of mutated sequences identified in all transfected cells. (**G**) The eight most prevalent resulting sequences in CFM_KO.

Immunofluorescence studies showed that the chicken fetal myoblasts (CFMs) we differentiated from primary cultures had a purity greater than 95% (Figure 1A). We designed single guide RNA (sgRNA) sequences targeting exon 1 of *MSTN*, designated as sgRNA1: TGATCAGTATGATGTCCAGA, and sgRNA2: TGTGATAATCGTCTCGGTTG; these two sgRNAs are separated by about 50bp in genome. We delivered the AdV-EGFP system and the AdV-EGFP-CRISPR system into the CFMs and into chicken embryonic fibroblasts (CEFs). After 48 hours of culture, the AdV-EGGP system produces wild-type (WT) cells, while the AdV-EGFP-CRISPR system produces knockout (KO) cells. These cells were divided into four groups: CFM_WT, CFM_KO, CEF_WT, and CEF_KO (Figure 1B–D).

Agarose gel electrophoresis of the WT cells demonstrated a 405-bp band for the *MSTN* PCR product, whereas the KO cells had two bands at 405 and about 351 bp (Figure 1E), indicating effective destruction of the targeted sequence of the *MSTN* gene in the KO cells. Detailed analysis of the mutations produced by CRISPR/Cas9 targeting showed that 75–80% of the sequence was mutated in both types of KO cells (Figure 1F). A strong preference (~53%) for perfect ligation of the cut sites was evident after CRISPR treatment (Figure 1G).

### 3.2. Differentially Expressed Genes (DEGs) between KO and WT CFMs

**Figure 2:**
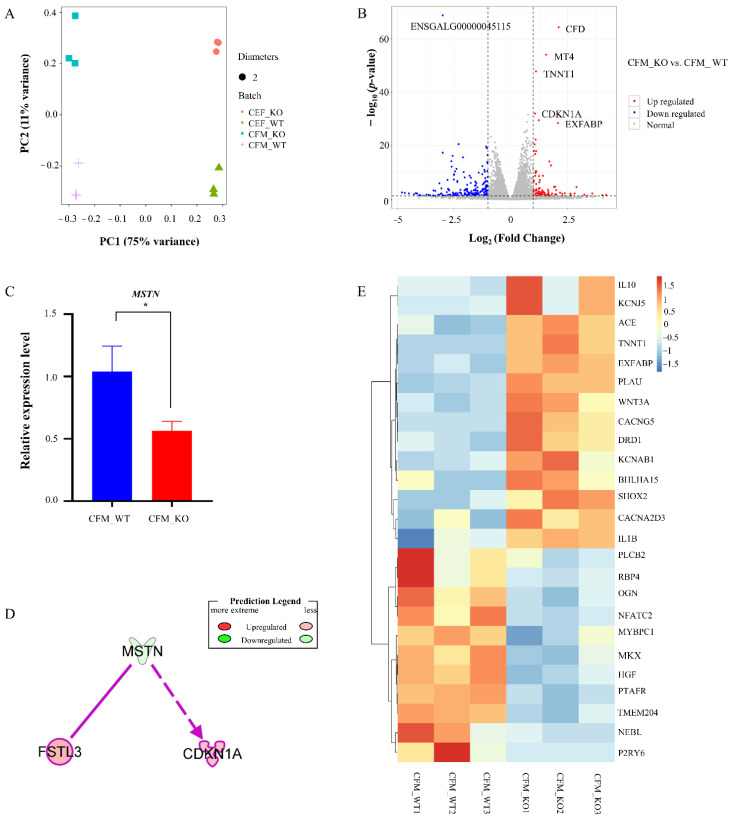
Differentially expressed genes (DEGs) analysis. (**A**) Principal component analysis for all primary chicken cells. Principal component 1 (PC1) and principal component 2 (PC2) were identified by logarithm transformation in DESeq2. (**B**) Volcano plot reveals significant DEGs in the comparison of CFM_KO vs. CFM_WT. (**C**) Expression level of *MSTN* in CFM_WT and CFM_KO groups detected by real-time PCR (* *p* < 0.05). (**D**) DEGs associated with *MSTN* in the CFM_KO vs. CFM_WT groups. (**E**) Heatmap hierarchical clustering revealed the DEGs related to skeletal muscle tissue development in CFM_KO vs. CFM_WT.

Transcriptome sequencing was used to detect the mRNA expression profiles of CFM_KO and CFM_WT. Each group contained 3 samples, and 6 libraries were constructed. An average of 31 million reads was generated for all the libraries, and more than 91% of the clean reads were mapped to the galGal6 chicken genome, suggesting good sequence quality. Principal component analysis (PCA) showed clear separation between CFM_KO and CFM_WT and concordance among group replicates (Figure 2A), indicating that the similarities between the samples in each group were high, and the two groups were completely separate.

Comparison of the CFM_KO and CFM_WT muscle cells revealed 296 significantly differentially expressed genes (DEGs), including 99 upregulated and 197 downregulated genes in the CFM_KO samples (Figure 2B, Table S2). The top ten DEGs are listed in Table 1. The expression of *MSTN* was downregulated in the CFM KO cells compared to the WT, and we also confirmed this result with real-time PCR (Figure 2C). Ingenuity Pathway Analysis (IPA) revealed an interaction between *follistatin like 3* (*FSTL3)* and *cyclin dependent kinase inhibitor 1A* (*CDKN1A)* with *MSTN* (Figure 2D). Furthermore, for 25 DEGs related to muscle development in CFMs, hierarchical clustering showed that those genes were related to skeletal muscle tissue development (Figure 2E). 

### 3.3. Enrichment Analysis of DEGs

**Figure 3:**
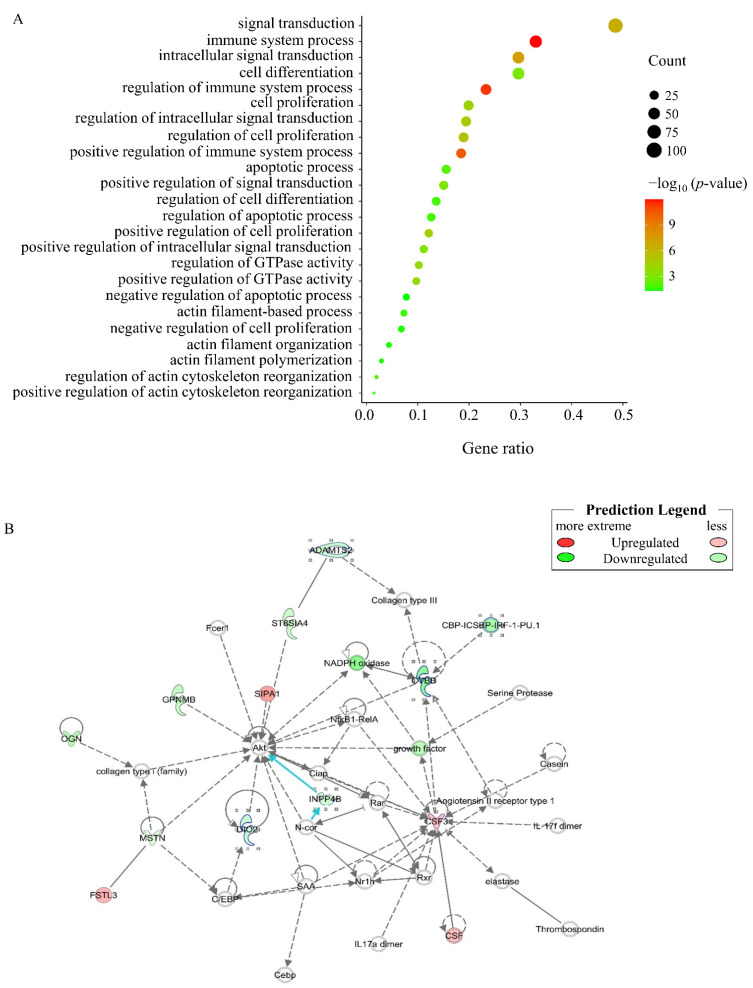
Representative enriched gene ontology (GO) terms and associated network of differentially expressed genes (DEGs) (CFM_KO vs. CFM_WT). (**A**) Representative enriched GO terms. (**B**) Gene network containing DEGs related to Connective Tissue Development and Function; Skeletal and Muscular System Development and Function; and Tissue Development.

Gene Ontology (GO) analysis performed on the *MSTN* KO cells to generate classification clusters identified 535 significantly enriched entries in the biological process category in the CFM_KO vs. CFM_WT comparison (Table S3). Most of the biological process-enriched items were significantly associated with cell proliferation and differentiation, muscle growth and development, signal transduction, apoptosis, the immune system, and energy metabolism (Figure 3A). Several major GO terms related to muscle contraction (positive regulation of actin cytoskeleton reorganization, regulation of actin cytoskeleton reorganization, actin filament polymerization, actin filament organization, and actin filament-based process) were also identified. 

The results of Kyoto Encyclopedia of Genes and Genomes (KEGG) pathway analyses revealed 27 pathways enriched with DEGs (Table S4), including the MAPK signaling pathway (*p* = 0.03) and the Pl3K/Akt pathway (*p* = 0.04). The canonical pathways were also identified by subjecting the DEGs to IPA (Table S5). Some muscle development-related pathways, such as FGF signaling, calcium signaling, and the STAT3 pathway, were enriched. FGF signaling is known to promote myoblast proliferation [23], calcium signaling is related to skeletal muscle development, maintenance, and regeneration [24], and the STAT3 pathway is a critical mediator of myoblast proliferation [25]. IPA analysis identified an *MSTN*-related differential gene network in CFM_KO cells that was associated with connective tissue development and function; skeletal and muscular system development and function; and tissue development (Figure 3B). As shown in Figure 3B, *MSTN* can act directly on Akt, or it can act on Akt through collagen type i or C/EBP family genes, such as *collagen type II alpha 1 chain (COL2A1)*, *collagen type XX alpha 1 chain* (*COL20A1)*, and *collagen type IV alpha 2 chain (COL4A2)*. Through enrichment analysis, we found some pathways, biological processes and networks related to muscle development, which deepened our understanding of the function and molecular mechanism of *MSTN* in birds.

### 3.4. Comparison of Transcriptome Changes after MSTN KO in CFMs and CEFs

**Figure 4:**
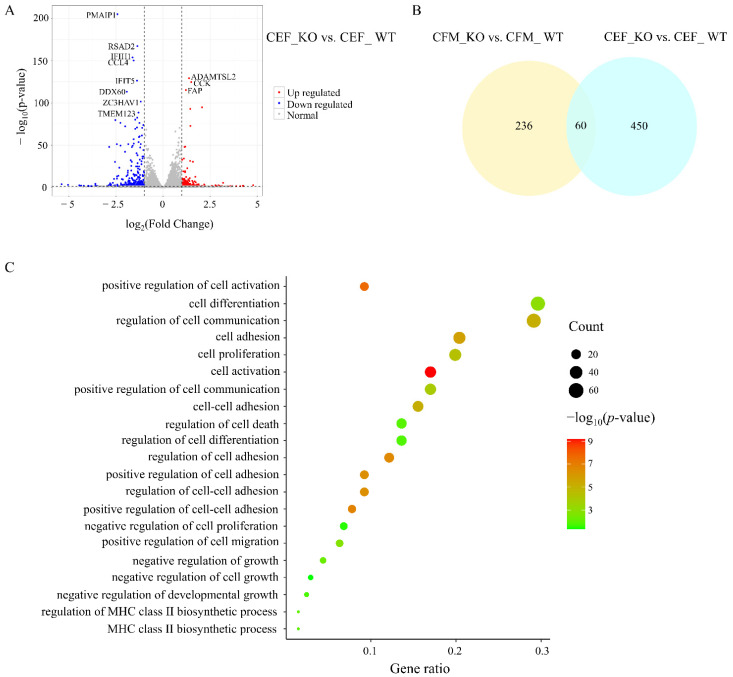
Differential gene analysis and enrichment analysis. (**A**) Volcano plot of significant differentially expressed genes (DEGs) of the CEF_KO vs. CEF_WT comparison. (**B**) A Venn diagram showing the DEGs identified from the CFM_KO vs. CFM_WT and the CEF_KO vs. CEF_WT comparisons. (**C**) The pathways were enriched only in CFM_KO vs. CFM_WT, but not in CEF_KO vs. CEF_WT.

The CEF_WT and CEF_KO cells each contained 3 cell libraries. PCA revealed clusters of similar samples in this experiment (Figure 2A). The two types of cells were completely separated, and the KO group and WT group of each cell line were completely separated. The CEFs samples showed 510 DEGs, including 178 upregulated genes and 332 downregulated genes in the CEF_KO samples (Figure 4A, Table S6). Comparison with the DEGs of the CFMs revealed 236 differential genes that were only expressed in muscle-related CFMs but not in embryonic CEFs (Figure 4B, Table S7). Among the 25 DEGs related to muscle development in CFMs, only 3 genes were also found in CEFs, namely *Potassium inwardly rectifying channel subfamily J member 5* (*KCNJ5)*, *interleukin 1, beta* (*IL1B)*, and *dopamine receptor D1* (*DRD1)*. The other 22 genes were unique to CFMs.

In total, 79 significantly enriched entries were identified in the biological process category in the CEF_KO vs. CEF_WT comparison (Table S8). The enrichment results for CEFs and CFMs indicated some common enrichment pathways (Figure S2). Comparison of the results for CEFs and CFMs revealed an enrichment of DEGs associated with the processes of cell differentiation, cell growth, cell adhesion, cell activation, and MHC class II biosynthesis in the CFMs (Figure 4C).

The results of KEGG pathway enrichment analysis revealed 35 pathways enriched with DEGs in the CEF_KO vs. CEF_WT comparison (Table S9). Cytokine-cytokine receptor interaction and NF-kappa B signaling pathway were present in the enrichment results of the two sets of DEGs. The MAPK signaling pathway was also enriched in CEF_KO cells (*p* = 0.09), but the Pl3K/Akt pathway was not enriched in CEF_KO cells. Using IPA to identify canonical pathways (Table S10), we identified three typical pathways related to muscle development in CFM_KO cells, but the STAT3 pathway was not enriched in CEF_KO cells.

## 4. Discussion

Muscle growth and development are usually regulated by specific core genes and signal transduction pathways [26,27]. Among the core genes, *MSTN* is the most important negative regulator [28]. The molecular mechanism of *MSTN* regulation in chicken muscle is not yet fully understood but understanding the potential regulatory mechanism of *MSTN* is very important in poultry meat production. Previous studies have used transcriptome analysis to explore the transcriptome profile of *MSTN* knockout (KO) muscle cells of mice and quails to understand the possible molecular mechanism, but few have explored the transcriptome profile of *MSTN* KO in chicken muscle cells [18,19]. Our aim in the present study was therefore to knock out *MSTN* in chicken muscle cells to allow a comparison of the transcriptome profiles of *MSTN* KO cells and WT cells. This approach systematically revealed DEGs, significantly enriched items, and KEGG pathways between the KO cells and WT cells, thereby providing reference data for the role of *MSTN* in muscle growth and development.

In the present research, transcriptome sequencing was used to analyze the DEGs between *MSTN* KO and WT CFMs. The comparison revealed *TNNT1* and *gap junction protein delta 2 (GJD2)* as DEGs under *MSTN* control specifically in chicken muscle cells, in agreement with previous mammalian *MSTN* knockout studies in mice and pigs [29,30]. *EXFABP* has also been found in *MSTN* KO chicken leg muscle [31]. Our study also identified several previously unreported DEGs that interacted with *MSTN* in chicken cells. One was *FSTL3*, a natural inhibitor of members of the TGF-β family, which mainly binds to *MSTN* to inhibit its function [32]. Another was *CDKN1A* (also known as *p21*), an *MSTN* target gene [33]. 

We also performed a similar *MTSN* knock out in chicken embryonic fibroblasts (CEFs), a model of embryonic development, to allow us to distinguish DEGs associated specifically with muscle growth from DEGs associated with general embryonic development. We found 236 DEGs that were only expressed in CFMs but not in CEFs. Of particular relevance was the finding that 22 of the 25 DEGs related to muscle development showed differential expression in CFMs but not in CEFs. Of those 22 muscle-related DEGs, *TNNT1* and *EXFABP* were the top DEGs in CFMs. Troponin T (TnT) is a core participant in the calcium regulation of actin filaments thin filament function, and *TNNT1* encodes slow skeletal muscle TnT [34]. *EXFABP* is a stress protein expressed during the differentiation of myoblasts [35] and may be essential in the differentiation of multinucleated myotubes [36]. *Myosin binding protein C, slow type* (*MYBPC1)* is associated with muscle structure [37], and the *ACE* gene controls skeletal muscle growth [38]. 

In mammals, *MSTN* generally functions through three signaling pathways: Smad activation, MAPK activation, and inhibition of Akt signaling [13]. The MAPK signaling pathway is a major regulator of skeletal muscle development [39], whereas the PI3K/AKT signaling pathway plays a key role in promoting cell proliferation and inhibiting cell death [40]. Knockout of *MSTN* in the muscle-related CFMs resulted in a significant enrichment of both the MAPK signaling pathway (*FGF16*, *CACNB4*, *FGF14*, *FGF19*, *CACNG5*, *IL1B*, *CACNA2D3*, *FGFR3*, and *FGF22*), and the PI3K-Akt signaling pathway (*CSF1R*, *COL2A1*, *SYK*, *COL4A2*, *ITGA4*, *HGF*, *IL2RG*, and *TLR4*). By contrast, in the CEFs, knockout of *MSTN* resulted only in enrichment of the MAPK pathway (*p* = 0.09), but not the PI3-Akt pathway. This enrichment result was consistent with the results previously reported for *MSTN* knockout in pig and mouse cells [30,41]. 

Our subsequent IPA analysis revealed the network and location where *MSTN* participates. The network diagram was related to connective tissue development and function; skeletal and muscular system development and function; and tissue development. *MSTN* can act directly on the Akt in the Pl3K/Akt pathway or indirectly through type i collagen or C/EBP. The IPA analysis also underscored the importance of the three main canonical pathways of DEGs in CFM_KO vs. CFM_WT involving muscle development—the FGF signaling pathway, calcium signaling, and STAT3 signaling pathway. Among these canonical pathways, the STAT3 pathway was not enriched in CEF_KO vs. CEF_WT, suggesting a specific involvement of the STAT3 pathway in muscle cell development. Interestingly, enrichment of the STAT3 signaling pathway was also reported in a *MSTN* knockout study in chicken leg muscles [31]. We speculate that this may be a unique regulatory pathway for *MSTN* in birds. These genes and pathways related to *MSTN* in this study provide a basis for molecular breeding to increase muscle production in commercial broilers.

## 5. Conclusions

In the present study, we knocked out the *MSTN* gene in CFMs and analyzed the DEGs between KO cells and WT cells to determine the role of *MSTN* specifically in muscle cells. We achieved about 50 bp deletions of *MSTN* in KO cells and clear downregulation of *MSTN* expression. Subsequent analysis revealed many genes that interact with or are affected by *MSTN*. Comparison of the transcriptome results of non-muscle CEFs with those of the muscle-related CFMs revealed the possible mechanism of action of *MSTN* in chicken muscle cells. Our study findings provide a valuable resource for understanding the biological functions and molecular mechanisms of *MSTN* for increasing muscle production in commercial meat chickens.

## Figures and Tables

**Table 1:** Top ten differentially expressed genes (DEGs) in CFM_KO vs. CFM_WT.

Gene Stable ID	Gene Name	Log2 Fold Change	*p* Value
ENSGALG00000045115		−2.998309986	1.38 × 10^−69^
ENSGALG00000040832	*CFD*	2.120508849	3.47 × 10^−65^
ENSGALG00000028451	*MT4*	1.569523444	6.71 × 10^−55^
ENSGALG00000029203	*TNNT1*	1.116180129	1.18 × 10^−48^
ENSGALG00000028318	*CDKN1A*	1.070316829	7.08 × 10^−33^
ENSGALG00000010741	*MAP1LC3C*	1.241659829	2.31 × 10^−30^
ENSGALG00000043064	*EXFABP*	2.087908822	2.64 × 10^−29^
ENSGALG00000006443	*MADPRT1*	1.103319425	3.97 × 10^−23^
ENSGALG00000044799	*VSIG4*	−2.295106641	2.13 × 10^−21^
ENSGALG00000053846		1.055098394	2.42 × 10^−21^

## Data Availability

The datasets presented in this study can be found in online repositories. The names of the repository/repositories and accession number(s) can be found below: http://www.ncbi.nlm.nih.gov/bioproject/776535, PRJNA776535 accessed on 30 January 2022.

## Supplementary Materials

The following are available online at https://www.mdpi.com/article/10.3390/genes13010058/s1, Figure S1: Schematic of the AdV-CRISPR system used in this study, Figure S2: Representative gene ontology (GO) enrichment terms of DEGs in the CFM_KO vs. CFM_WT groups and CEF_KO vs. CEF_WT, Table S1: Prime list, Table S2: The differentially expressed genes in CFM_KO vs. CFM_WT, Table S3: GO enrichment terms of DEGs in the CFM_KO vs. CFM_WT groups, Table S4: KEGG enrichment terms of DEGs in the CFM_KO vs. CFM_WT groups, Table S5: Canonical pathways about cellular growth, proliferation, and development in the CFM_KO vs. CFM_WT, Table S6: The differentially expressed genes in CEF_KO vs. CEF_WT, Table S7: The differential genes that were only expressed in CFM_KO vs. CFM_WT, Table S8: GO enrichment terms of DEGs in the CEF_KO vs. CEF_WT groups, Table S9: KEGG enrichment terms of DEGs in the CEF_KO vs. CEF_WT groups, Table S10: Canonical pathways about cellular growth, proliferation, and development in the CEF_KO vs. CEF_WT.

## References

[1] Doran T.J. (2016). Advances in genetic engineering of the avian genome: “Realising the promise”. Transgenic Res..

[2] Kim S.W., Lee J.H., Park B.C., Park T.S. (2017). Myotube differentiation in clustered regularly interspaced short palindromic repeat/Cas9-mediated MyoD knockout quail myoblast cells. Asian-Australas. J. Anim. Sci..

[3] McPherron A.C., Lawler A.M., Lee S.J. (1997). Regulation of skeletal muscle mass in mice by a new TGF-beta superfamily member. Nature.

[4] Lee S.J. (2004). Regulation of muscle mass by myostatin. Annu. Rev. Cell Dev. Biol..

[5] Kambadur R., Sharma M., Smith T.P., Bass J.J. (1997). Mutations in myostatin (GDF8) in double-muscled Belgian Blue and Piedmontese cattle. Genome Res..

[6] Cyranoski D. (2015). Super-muscly pigs created by small genetic tweak. Nature.

[7] Kim Y.S., Bobbili N.K., Paek K.S., Jin H.J. (2006). Production of a monoclonal anti-myostatin antibody and the effects of in ovo administration of the antibody on posthatch broiler growth and muscle mass. Poult. Sci..

[8] Kim G.D., Lee J.H., Song S.M., Kim S.W., Han J.S., Shin S.P., Park B.C., Park T.S. (2020). Generation of myostatin-knockout chickens mediated by D10A-Cas9 nickase. Faseb J..

[9] He Z.Y., Zhang T., Jiang L., Zhou M.Y., Wu D.J., Mei J.Y., Cheng Y. (2018). Use of CRISPR/Cas9 technology efficiently targetted goat myostatin through zygotes microinjection resulting in double-muscled phenotype in goats. Biosci. Rep..

[10] Schuelke M., Wagner K.R., Stolz L.E., Hubner C., Riebel T., Komen W., Braun T., Tobin J.F., Lee S.J. (2004). Myostatin mutation associated with gross muscle hypertrophy in a child. N. Engl. J. Med..

[11] Yang W., Chen Y., Zhang Y., Wang X., Yang N., Zhu D. (2006). Extracellular signal-regulated kinase 1/2 mitogen-activated protein kinase pathway is involved in myostatin-regulated differentiation repression. Cancer Res..

[12] Elkasrawy M.N., Hamrick M.W. (2010). Myostatin (GDF-8) as a key factor linking muscle mass and bone structure. J. Musculoskel. Neuron. Interact..

[13] Elkina Y., von Haehling S., Anker S.D., Springer J. (2011). The role of myostatin in muscle wasting: An overview. J. Cachexia Sarcopeni. Muscle.

[14] Paswan C., Bhattacharya T., Nagaraj C., Chaterjee R., Jayashankar M. (2014). SNPs in minimal promoter of myostatin (GDF-8) gene and its association with body weight in broiler chicken. J. Appl. Anim. Res..

[15] Zhang G., Zhao X., Wang J., Ding F., Zhang L. (2012). Effect of an exon 1 mutation in the myostatin gene on the growth traits of the Bian chicken. Anim. Genet..

[16] Lee J., Kim D.H., Lee K. (2020). Current Approaches and Applications in Avian Genome Editing. Int. J. Mol. Sci..

[17] Li T.T., Zhang G.X., Wu P.F., Duan L., Li G.H., Liu Q.H., Wang J.Y. (2018). Dissection of Myogenic Differentiation Signatures in Chickens by RNA-Seq Analysis. Genes.

[18] Huang P., Pang D., Wang K., Xu A., Yao C., Li M., You W., Wang Q., Yu H. (2019). The Possible Role of Complete Loss of Myostatin in Limiting Excessive Proliferation of Muscle Cells (C2C12) via Activation of MicroRNAs. Int. J. Mol. Sci..

[19] Park J.W., Lee J.H., Han J.S., Shin S.P., Park T.S. (2020). Muscle differentiation induced by p53 signaling pathway-related genes in myostatin-knockout quail myoblasts. Mol. Biol. Rep..

[20] Sato F., Kurokawa M., Yamauchi N., Hattori M. (2006). Gene silencing of myostatin in differentiation of chicken embryonic myoblasts by small interfering RNA. Am. J. Physiol.-Cell Physiol..

[21] Yi X., Tao Y., Lin X., Dai Y., Yang T.L., Yue X.J., Jiang X.J., Li X.Y., Jiang D.S., Andrade K.C. (2017). Histone methyltransferase Setd2 is critical for the proliferation and differentiation of myoblasts. Bba-Mol. Cell Res..

[22] Ringnér M. (2008). What is principal component analysis?. Nat. Biotechnol..

[23] Vishal K., Lovato T.L., Bragg C., Chechenova M.B., Cripps R.M. (2020). FGF signaling promotes myoblast proliferation through activation of wingless signaling. Dev. Biol..

[24] Tu M.K., Levin J.B., Hamilton A.M., Borodinsky L.N. (2016). Calcium signaling in skeletal muscle development, maintenance and regeneration. Cell Calcium.

[25] Zhang C.C., Li Y.L., Wu Y.N., Wang L.Y., Wang X.N., Du J. (2013). Interleukin-6/Signal Transducer and Activator of Transcription 3 (STAT3) Pathway Is Essential for Macrophage Infiltration and Myoblast Proliferation during Muscle Regeneration. J. Biol. Chem..

[26] Wu P.F., Dai G.J., Chen F.X., Chen L., Zhang T., Xie K.Z., Wang J.Y., Zhan G.X. (2018). Transcriptome profile analysis of leg muscle tissues between slow- and fast-growing chickens. PLoS ONE.

[27] Ayuso M., Fernandez A., Nunez Y., Benitez R., Isabel B., Barragan C., Fernandez A.I., Rey A.I., Medrano J.F., Canovas A. (2015). Comparative Analysis of Muscle Transcriptome between Pig Genotypes Identifies Genes and Regulatory Mechanisms Associated to Growth, Fatness and Metabolism. PLoS ONE.

[28] Parsons S.A., Millay D.P., Sargent M.A., McNally E.M., Molkentin J.D. (2006). Age-dependent effect of myostatin blockade on disease severity in a murine model of limb-girdle muscular dystrophy. Am. J. Pathol..

[29] Braga M., Pervin S., Norris K., Bhasin S., Singh R. (2013). Inhibition of In Vitro and In Vivo Brown Fat Differentiation Program by Myostatin. Obesity.

[30] Li X., Xie S.S., Qian L.L., Cai C.B., Bi H.F., Cui W.T. (2020). Identification of genes related to skeletal muscle growth and development by integrated analysis of transcriptome and proteome in myostatin-edited Meishan pigs. J. Proteom..

[31] Xu K., Han C.X., Zhou H., Ding J.M., Xu Z., Yang L.Y., He C., Akinyemi F., Zheng Y.M., Qin C. (2020). Effective MSTN Gene Knockout by AdV-Delivered CRISPR/Cas9 in Postnatal Chick Leg Muscle. Int. J. Mol. Sci..

[32] Brandt C., Hansen R.H., Hansen J.B., Olsen C.H., Galle P., Mandrup-Poulsen T., Gehl J., Pedersen B.K., Hojman P. (2015). Over-expression of Follistatin-like 3 attenuates fat accumulation and improves insulin sensitivity in mice. Metabolism.

[33] Ohsawa Y., Hagiwara H., Nakatani M., Yasue A., Moriyama K., Murakami T., Tsuchida K., Noji S., Sunada Y. (2006). Muscular atrophy of caveolin-3-deficient mice is rescued by myostatin inhibition. J. Clin. Invest..

[34] Wei B., Jin J.P. (2016). TNNT1, TNNT2, and TNNT3: Isoform genes, regulation, and structure-function relationships. Gene.

[35] Cancedda F.D., Dozin B., Zerega B., Cermelli S., Cancedda R. (2001). Extracellular fatty acid binding protein (ex-FABP) is a stress protein expressed during chondrocyte and myoblast differentiation. Osteoarthr. Cartil..

[36] Gentili C., Cermelli S., Tacchetti C., Cossu G., Cancedda R., Cancedda F.D. (1998). Expression of the extracellular fatty acid binding protein (Ex-FABP) during muscle fiber formation in vivo and in vitro. Exp. Cell Res..

[37] Kong B.W., Hudson N., Seo D., Lee S., Khatri B., Lassiter K., Cook D., Piekarski A., Dridi S., Anthony N. (2017). RNA sequencing for global gene expression associated with muscle growth in a single male modern broiler line compared to a foundational Barred Plymouth Rock chicken line. BMC Genom..

[38] Davis R.V.N., Lamont S.J., Rothschild M.F., Persia M.E., Ashwell C.M., Schmidt C.J. (2015). Transcriptome Analysis of Post-Hatch Breast Muscle in Legacy and Modern Broiler Chickens Reveals Enrichment of Several Regulators of Myogenic Growth. PLoS ONE.

[39] Keren A., Tamir Y., Bengal E. (2006). The p38 MAPK signaling pathway: A major regulator of skeletal muscle development. Mol. Cell Endocrinol..

[40] Chelh I., Meunier B., Picard B., Reecy M.J., Chevalier C., Hocquette J.F., Cassar-Malek I. (2009). Molecular profiles of Quadriceps muscle in myostatin-null mice reveal PI3K and apoptotic pathways as myostatin targets. BMC Genom..

[41] Wang L.M., Huang Y., Wang X.L., Chen Y.L. (2019). Label-Free LC-MS/MS Proteomics Analyses Reveal Proteomic Changes Accompanying MSTN KO in C2C12 Cells. Biomed. Res. Int..

