# Supplementary material for: Transcriptomic Analysis of MSTN Knockout in the Early Differentiation of Chicken Fetal Myoblasts
Source: Genes (Basel). 2021 Dec 26;13(1):58. doi: 10.3390/genes13010058 (PMC8774668; doi:10.3390/genes13010058)
Supplement: Supplementary file 1 [file genes-13-00058-s001.zip › Supplementary Material.pdf]

Supplementary Material

Transcriptomic analysis of *MSTN* knockout in the early differentiation of chicken fetal myoblasts

Ke Xu [1], Hao Zhou [1], Chengxiao Han [1], Zhong Xu [2], Jinmei Ding [1], Jianshen Zhu [1], Chao Qin [1], Huaixi Luo [1], Kangchun Chen [1], Jiajia Liu [1], Wenqi Zhu [1] and He Meng [1,*]

1 Shanghai Key Laboratory of Veterinary Biotechnology, Department of Animal Science, School of Agriculture and Biology, Shanghai Jiao Tong University, Shanghai, China; keristina@sjtu.edu.cn
2 Hubei Key Laboratory of Animal Embryo and Molecular Breeding, Institute of Animal Husbandry and Veterinary, Hubei Provincial Academy of Agricultural Sciences, Wuhan, China; 642205305@qq.com
* Correspondence: menghe@sjtu.edu.cn

Supplementary Figures

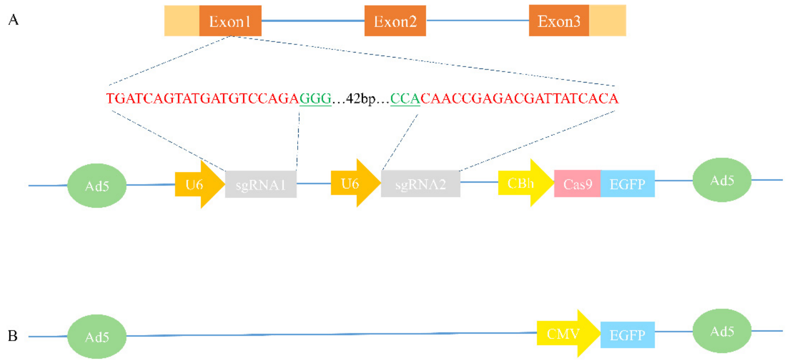

Figure S1. Schematic of the AdV-CRISPR system used in this study. (**A**) The AdV-EGFP-CRISPR system expressing EGFP, SpCas9 and a pair of sgRNAs. The two sgRNA are about 50bp apart in the genome. (**B**) The AdV-EGFP system only expresses EGFP.

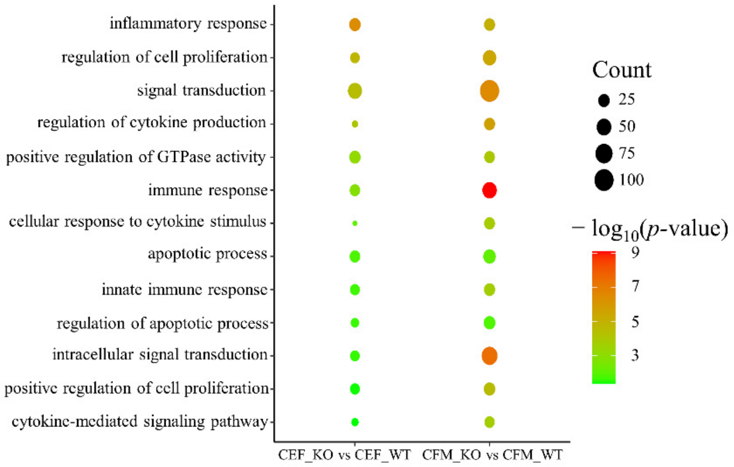

Figure S2. Representative gene ontology (GO) enrichment terms of DEGs in the CFM_KO vs. CFM_WT groups and CEF_KO vs. CEF_WT.
